# Rates and risk factors for drug resistance tuberculosis in Northeastern China

**DOI:** 10.1186/1471-2458-13-1171

**Published:** 2013-12-13

**Authors:** Qiao Liu, Limei Zhu, Yan Shao, Honghuan Song, Guoli Li, Yang Zhou, Jinyan Shi, Chongqiao Zhong, Cheng Chen, Wei Lu

**Affiliations:** 1 Department of Chronic Communicable Disease, Center for Disease Control and Prevention of Jiangsu Province, 172 Jiangsu Rd, Nanjing 210009, PR China; 2 Department of Chronic Communicable Disease, Center for Disease Control and Prevention of Lianyungang City, Lianyungang, PR China

**Keywords:** Epidemiology, MDR-TB, Drug resistance, Drug susceptibility

## Abstract

**Background:**

Drug-resistant tuberculosis (TB) has emerged as a major challenge toward TB control and prevention. In Lianyungang city, the extent and trend of drug resistant TB is not well known. The objective of the survey was to assess drug resistance pattern of MTB and risk factors for drug resistant TB, including multidrug resistance tuberculosis (MDR-TB) in this area.

**Methods:**

We performed drug susceptibility testing on *Mycobacterium tuberculosis* (MTB) isolates with first- and second-line anti-tuberculosis drugs of 1012 culture positive TB cases by using the proportion method, who were consecutively enrolled from January 2011 to December 2012 in Lianyungang city, China. The patterns of drug resistance in MTB were investigated and multiple logistic regression analysis was performed to assess the risk factors for drug resistant TB.

**Results:**

Among the 1012 strains tested, 308 (30.4%) strains were resistant to at least one first-line drug; the prevalence of MDR-TB was 88 (8.7%), 5 (0.5%) strains were found to be extensively drug-resistant tuberculosis (XDR-TB). Female gender was a risk factor for MDR-TB (adjusted odds ratio (aOR) 1.763, 95% CI (1.060-2.934). The aged 28–54 years was significantly associated with the risk of MDR-TB with an aOR: 2.224, 95% CI (1.158-4.273) when compared with those 65 years or older. Patients with previous treatment history had a more than 7-fold increased risk of MDR-TB, compared with those never previously treated.

**Conclusions:**

The burden of drug resistant TB cases is sizeable, which highlights an urgent need to reinforce control, detection and treatment strategies for drug resistant TB.

## Background

Tuberculosis (TB) remains a major global health problem, especially in high TB burden countries with large numbers of TB patients and poorer sanitation. TB ranks as the second leading cause of death among infectious diseases worldwide, following only the human immunodeficiency virus (HIV) [1]. According to the 2012 WHO global TB report, China ranks as 2nd among the world’s 22 high burden countries with a TB incidence around 1 million, and the prevalence rate of TB was 104 per 100,000 patients (95% confidence interval [95% CI] 91–119) and the incidence rate was 75 per 100,000 patients (95% CI 66–85) in 2011 [1].

In a nationwide survey in China in 2007, the estimated multidrug-resistant tuberculosis (MDR-TB) rate was 5.7% for new cases and 25.6% for previously treated cases. Approximately 8% of the patients with MDR-TB had extensively drug-resistant tuberculosis (XDR-TB) [2]. However, information on the prevalence of MDR and XDR-TB remains scant in the region. Drug-resistant *Mycobacterium tuberculosis*(MTB) strains, MDR-TB and XDR-TB strains may be the potential propellers for the spread of TB, and through now we put great efforts in TB control and treatment, the situation has not greatly improved. Despite poor compliance to chemotherapy duration, drug resistance of MTB would be another important factor for treatment failure among new TB cases [3], which may lead to a fall in successful TB cure rates [4].

Lianyungang city is located in the northeastern part of China, and covers an area of 7.5 thousand square kilometers with a population around 5 million in 2012. Information on the prevalence of MDR-TB and XDR-TB remains unknown in this region. Thus, we conducted this study to assess drug resistance patterns of MTB on first- and second-line anti-TB drugs in this region, and evaluated related risk factors of drug resistance among new and previously treated smear-positive TB patients.

## Methods

### Study population and isolates

In Lianyungang city, all newly registered patients with sputum smear-positive pulmonary tuberculosis were collected for strain identification and drug susceptibility testing (DST). From January 2011 to December 2012, a total of 1170 clinical isolates were collected from the sputum samples of pulmonary tuberculosis patients. In all surveys, each newly registered TB patient, positive on sputum smear microscopy, was interviewed by the clinician using the medical records to obtain the treatment history. The medical records include personal information, physical examinations, present illness, TB-related complaints, previous medical history, family history and PPD result. The treatment history of included cases was classified by medical staff into new and previously treated cases. New cases were defined as patients with tuberculosis who have never been treated with anti-TB drugs or received them for less than one month. Previously treated cases were defined as patients who have been treated for tuberculosis for at least one month. The definitions of new/previously treated cases referred to the WHO guidelines [5].

### Strain identification and drug susceptibility test

The sputum samples were cultured and isolated on Lowenstein-Jensen (LJ) culture media which were prepared by the Baso biotechnology Ltd (Zhuhai, China). All the MTB isolates from positive culture were identified and subjected to DST. Identification of MTB was done using the p-Nitrobenzoic acid (PNB) method and the growth in LJ media containing PNB indicates that the bacilli do not belong to the MTB complex. Species other than MTB were excluded from the final analysis.

LJ media impregnated one anti-tuberculosis drug was used for DST, and the corresponding drug concentrations were as follows: 0.2 μg/ml for INH, 40 μg/ml for RMP, 4 μg/ml for SM, 2 μg/ml for EMB, 30 μg/ml for KM and 2 μg/ml for OFX. The LJ culture media were incubated at 37°C.They were read twice at the first week to detect contaminations and/or fast growth of atypical mycobacteria and again every week thereafter for slower growing bacteria; if no bacteria grew by 6 weeks, the result was recorded as negative. Contaminated slant cultures were discarded, and additional decontamination and culture were undertaken using a stored portion of the original sample. Resistance was expressed as the percentage of colonies that grew on the drug-containing media compared to those on control media. The growth of colonies in the drug containing plate was compared to the control plate as a proportion. If the bacterial growth on the media with the specific drug was ≥1% compared to the control, the strain was declared resistant to the specific drug; or it was defined as sensitive when the growth rate was < 1% compared to the control. Strains isolation, identification and DST were performed at the fourth people’s hospital of Lianyungang city. Procedures for external quality assurance for smear and culture were based on WHO guidelines [1]. For internal quality assurance of DST, a standard H37Rv strain was included with each new batch of LJ media. External quality control for culture and DST was conducted by the provincial TB reference laboratory, which participates in the annual proficiency review of DST organized by the Hong Kong Supranational Tuberculosis Reference Laboratory and has passed each review since 2010.

### MDR and XDR

The following WHO-recommended definitions were used for resistant cases:

MDR-TB was defined as TB with resistance to at least INH and RMP. XDR-TB defined as MDR-TB plus resistance to a fluoroquinolone and at least one second-line injectable agent: amikacin, kanamycin and/or capreomycin [5]. In our study the XDR-TB was defined as TB with resistance to at least INH, RMP, KM and OFX.

### Statistical analysis

For comparison of categorical variables, significance testing was done by χ2 test with by 2-sided Fisher exact test as appropriate. Associations between selected factors were estimated by computing odds ratios (ORs) and their 95% confidence intervals (CIs) from an unconditional logistic regression model. The criterion for significance was set at *P* < 0.05 based on a two-sided test. Analyses were conducted with SPSS version 13.0 (SPSS Inc., Chicago, IL, USA).

### Ethics statement

Informed written consent was obtained from all patients and the study was approved by the Institutional Review Board of Jiangsu Provincial Center for Disease Control and Prevention. Ethics were respected throughout the study period.

## Results

### Bacterial strains and patient characteristics

**Figure 1:**
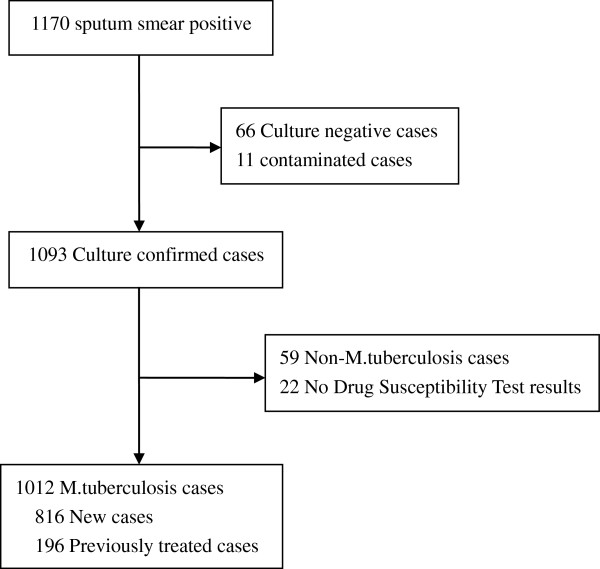
Flow diagram of tuberculosis subjects included in this study.

During the study period, 1170 sputum smear positive TB cases were enrolled and 1093 clinical isolates reported a culture positive MTB. Meanwhile, 66 cultured negative cases and 11 contaminated cases were excluded. Of the 1093 culture positive samples, 59 cultures were distinguished as Non-MTB (NTM). 22 samples failed for DST assay were excluded. Thus, 1012 samples were included in the final analysis (Figure 1). The age of the 1012 patients ranged from 14 to 90 years (mean ± SD), 49.8 ± 20.6, media 54), 748 (73.9%) of them were male and 264 (26.1%) were female. Of the 1012 case-patients included in the study, 816 (80.6%) were new incidence and 196 (19.4%) were previously treated. 9 (0.9%) cases were migrants.859 (84.9%) of the 1012 cases came from rural areas (Table 1).

**Table 1:** Characteristics of Patients with Culture Confirmed Pulmonary Tuberculosis: comparison of patients with any drug resistance, MDR-TB and XDR-TB

**Variable**	**Totally 1012,n(%)**	**Any drug resistance, n(%)**	***p*-Value**	**MDR-TB, n(%)**	***p*-Value**	**XDR-TB, n(%)**	***p*-Value**
**Sex**							
Female	264(26.1)	89(33.7)	0.178	32(12.1)	0.022	3(1.1)	0.115*
Male	748(73.9)	219(29.3)		56(7.5)		2(0.3)	
**Age groups (years)**							
0-28	250(24.7)	44(17.6)	<0.001	13(5.2)	0.002	1(0.4)	0.208*
28-54	254(25.1)	94(37.0)		35(13.8)		1(0.4)	
54-65	230(22.7)	82(35.7)		23(10.0)		3(1.3)	
65+	278(27.5)	88(31.7)		17(6.1)		0(0)	
**Migrant population**							
Yes	9(0.9)	6(66.7)	0.027*	2(22.2)	0.181*	0(0)	1*
No	1003(99.1)	302(30.1)		86(8.6)		5(0.5)	
**Treatment history**							
New Cases	816(80.6)	191(23.4)	<0.001	34(4.2)	<0.001	0(0)	<0.001*
Pre Treated Cases	196(19.4)	117(59.7)		54(27.6)		5(2.6)	
**Region**							
Rural areas	859(84.9)	258(30.0)	0.512	69(8.0)	0.076	3(0.3)	0.167*
Urban Districts	153(15.1)	50(32.7)		19(12.4)		2(1.3)	
**Occupation**							
Farmer	783(77.4)	247(31.5)	0.156	69(8.8)	0.808	1(0.4)	1*
Non-farmer	229(22.6)	61(26.6)		19(8.3)		4(0.5)	

*Value by Fisher’s exact test.

### Prevalence of resistance to anti-tuberculosis drugs

This study observed most resistance of isoniazid 184 (18.2%) followed by streptomycin 141 (13.9%), rifampicin 114 (11.3%), ofloxacin 78 (7.7%), ethambutol 72 (7.1%), and kanamycin 42 (4.2%). Among the new cases, drug resistance was reported in 191(23.4%); INH resistance was reported in 91 (11.2%), RMP resistance in 48 (5.9%), EMB resistance in 30 (3.7%), SM resistance in 90 (11.0%), KM resistance in 28 (3.4%), OFX resistance in 42 (5.1%), Mono-resistance was found in 113 patients (13.8%), MDR in 34 (4.2%) and XDR in 0 (0%). Among the 196 previously treated cases, drug resistance was reported in 117 (59.7%); INH resistance was reported in 93 (47.4%), RMP resistance in 66 (33.7%), EMB resistance in 42 (21.4%), SM resistance in 51 (26.0%), KM resistance in 14 (7.1%), OFX resistance in 36 (18.4%), Mono-resistance was present in 22 (11.2%), MDR in 54 (27.6%) and XDR in 5 (2.6%) patients, respectively (Table 2).

**Table 2:** Prevalence of anti-tuberculosis drug resistance among M. tuberculosis isolates from new and previously treated patients the results of drug susceptibility pattern by type of case

	**New cases n (%)**	**Previously treated n (%)**	**Combined n (%)**
**Total strans tested**	816	196	1012
**Susceptibility to all drugs**	625(76.6)	79(40.3)	704(69.6)
Susceptibility to fist-line	661(81.0)	85(43.4)	746(73.7)
Susceptibility to second-line	751(92.0)	155(79.1)	906(89.5)
**Any resistance**	191(23.4)	117(59.7)	308(30.4)
H	91(11.2)	93(47.4)	184(18.2)
R	48(5.9)	66(33.7)	114(11.3)
E	30(3.7)	42(21.4)	72(7.1)
S	90(11.0)	51(26.0)	141(13.9)
K	28(3.4)	14(7.1)	42(4.2)
O	42(5.1)	36(18.4)	78(7.7)
**Mono-resistance**	113(13.8)	22(11.2)	135(13.3)
H only	27(3.3)	8(4.1)	35(3.5)
R only	11(1.3)	3(1.5)	14(1.4)
E only	1(0.1)	0	1(0.1)
S only	42(5.1)	5(2.6)	47(4.6)
K only	18(2.2)	2(1.0)	20(2.0)
O only	14(1.7)	4(2.0)	18(1.8)
**MDR-TB**	34(4.2)	54(27.6)	88(8.7)
H + R	5(0.6)	13(6.6)	18(1.8)
H + R + E	2(0.2)	5(2.6)	7(0.7)
H + R + S	7(0.9)	10(5.1)	17(1.7)
H + R + K	0	0	0
H + R + O	2(0.2)	5(2.6)	7(0.7)
H + R + E + S	7(0.9)	4(2.0)	11(1.1)
H + R + E + K	0	0	0
H + R + E + O	4(0.5)	4(2.0)	8(0.8)
H + R + S + K	1(0.1)	1(0.5)	2(0.2)
H + R + S + O	2(0.2)	1(0.5)	3(0.3)
H + R + K + O	0	1(0.5)	1(0.1)
H + R + E + S + K	0	0	0
H + R + E + S + O	4(0.5)	6(3.1)	10(1.0)
H + R + E + K + O	0	1(0.5)	1(0.1)
H + R + S + K + O	0	1(0.5)	1(0.1)
H + R + E + S + K + O	0	2(1.0)	2(0.2)
**XDR-TB**	0	5(2.6)	5(0.5)

H: isoniazid, R: rifampicin, E: ethambutol, S: streptomycin, K: kanamycin and O: ofloxacin. MDR-TB defined as resistance to at least H and R. XDR-TB defined as resistance to at least H, R, K and O.

### Factors associated with MDR-TB

The univariate analysis revealed a higher proportion of female gender (cOR 1.704, 95% CI 1.077–2.697), individuals aged 28–54 years (cOR 2.914, 95% CI 1.502–5.652), and patients with previous TB treatment (cOR 8.746, 95% CI 5.495–13.922) amongst MDR-TB patients. The final multivariable logistic regression model identified female gender (aOR 1.763, 95% CI 1.060–2.934), aged 28–54 years (aOR 2.224, 95% CI 1.158–4.273) and previous history of TB treatment (aOR 8.910, 95% CI 5.478–14.492), to be associated with MDR-TB (Table 3).

**Table 3:** Factors associated with multi-drug resistant tuberculosis

**Variable**	**Non-MDR, 924**	**MDR-TB, 88**	**Univariate**			**Multivariate**		
	**n(%)**	**n(%)**	**cOR**	**95% CI**	***p*-Value**	**aOR**	**95% CI**	***p*-Value**
Female gender	232(25.1)	32(36.4)	1.704	1.077–2.697	0.022	1.763	1.060–2.934	**0.029**
Age 28–54 yr	219(23.7)	35(39.8)	2.914	1.502–5.652	0.002	2.224	1.158–4.273	**0.016**
migrant population	7(0.8)	2(2.3)	3.047	0.623–14.894	0.181*	4.385	0.807–23.821	0.087
Previous TB treatment	142(15.4)	54(61.4)	8.746	5.495–13.922	<0.001	8.910	5.478–14.492	**<0.001**
Rural areas	790(85.5)	69(78.4)	0.616	0.359–1.057	0.076	0.583	0.321–1.058	0.076

cOR, crude odds ratio; aOR, adjusted odds ratio, adjusted for sex, age, migrant population, treatment history and region. Values in boldface indicate a significant (*P*<0.05) difference between Non-MDR and MDR-TB.

## Discussion

Information on anti-tuberculosis drug resistance levels is an essential management tool for evaluating the performance of national TB control programmers (NTPs). Resistance in previously treated cases is an indicator of current treatment practices in the community. Drug resistance in new cases reflects transmission of disease with resistant bacilli [6].

The prevalence of drug resistance in previously treated cases was higher than new cases for each drug alone as well as for all six drugs, as reported previously [2,7,8]. This suggests that retreatment is deficient and poses a threat to continued transmission, which has not yet manifested itself among new patients. The prevalence of MDR-TB was 4.2% in new cases and 27.6% in previously treated cases. As reported in a national survey of China, the rates for MDR-TB in new and previously treated cases were 5.7% and 25.6%, respectively [2]. The MDR-TB prevalence among previously treated cases was modestly higher than the national average, and among new cases the rate was slightly lower. MDR-TB was observed 2.8% -14.7% of new cases and 9.7% -34.3% of previously treated cases in China [2,9-12]. In Korea, resistance to at least one first-line drug was identified in 11.7% of new cases and 41.6% of previously treated cases. MDR-TB was detected in 3.9% of new cases and 27.2% of previously treated cases. The proportion of XDR-TB among MDR-TB patients was 16.7% (9/54) [13]. In Japan, The prevalence of MDR in new and previously treated cases was 0.7% and 9.8% respectively [14]. The overall MDR-TB rate was 4.3%; 2.5% in new cases and 13.9% in previously treated cases in Pakistan [15]. MDR-TB was 0.2% of new cases and 3.4% of previously treated cases in Madagascar [16]. In Cambodia, no single MDR-TB was found among the new cases and 3.1% in previously treated cases [17]. In Yemen, The prevalence of MDR in new and previously treated cases was 3% and 9.4% respectively [18].

Compared MDR-TB patients, patients with XDR-TB are more likely to die or have treatment failure [19,20]. The prevalence of XDR-TB among combined cases was 0.5%; it was 0% and 2.6% (5 cases) in new and previously treated cases, respectively. XDR-TB was distributed widely, albeit sporadically. This might imply that China’s treatment strategy has improved, but that community TB control is still insufficient [21]. The prevalence of XDR-TB in new cases was slightly lower than the national average (0.5%). However, XDR-TB prevalence among previously treated cases was a little higher than the national average (2.1%) [2]. These findings points to the need for interventions that will increase continuity of treatment and reduce the rate of treatment default, especially among patients treated within the hospital system [22].

As reported, 11% of new cases of tuberculosis and 16% of previously treated cases were resistant to either INH or RMP (but not both) before they received standard first-line short-course treatment [2]. Resistance to these first-line drugs were usually not detected because culturing and drug susceptibility testing are not routinely performed at the local tuberculosis clinics. The use of standard first-line drugs in the treatment of these patients may increase the risk of relapse, treatment failure and acquired drug resistance [23-25].

Male or female TB patients could have different levels of risk for drug resistance due to differences in access to health-care services or exposure to other risk factors [5]. In the present study, the resistance rate to any drug was similar in female and male, but females were more likely to have MDR-TB than males, with an OR of 1.763 (95% CI: 1.060-2.934), also reported in previous studies [8,26-29]. The reasons for the association between female gender and MDR-TB are not well known. We hypothesize that this association could be related to the fact that women spend a long period of time caring for men and others with MDR-TB both in households and in healthcare settings in China where the majority of health care workers are female [26,27]. TB diagnosis and DST may be delayed among female patients, making treatment more difficult and inappropriate chemotherapeutic regimens more likely [30,31]. It is also possible that referring patients to the hospital may be different for male and female patients and the providers may feel reluctant to refer female patients with less complicated infection to a tertiary care center [32]. Discovering gender disparities associated with the risks of MDR-TB could provide insight into the development of targeted measures and improve access to health care and reduce the risk of acquiring drug-resistance.

We also found that age was a significant factor in the development of drug resistance. Resistance to any drug and MDR was highest in those aged 28–54 years. The association between age and the risk of MDR-TB is not well established in the literature as different studies use different cut-off points for age groups. However, it was also reported that MDR-TB patients were more likely to be younger than 65 years [33,34]. We assumed that age-related difference in treatment adherence might be a possible explanation, as patients at 28–54 years old were often occupied by study, work or other activities on a daily basis, in contrast with the more sedentary lifestyle of elderly patients [34].

Many other risk factors for drug resistance tuberculosis and MDR-TB have been identified in recent studies: such as irregular treatment [35], urban residence [12], non-permanent residents [34], urban migration [12], lack of a sewage system in the home [35], frequent travelers [34], alcoholism plus smoking, and lung cavities [35]. Other important risk factors are associated with the supply or quality of the drugs, the possible inadequate drug intake by patients, and others, such as the lack of full supervision during the intensive phase of treatment [36], poor NTPs [14,17], and the possible high prevalence of highly virulent MDR-TB strains of MTB [17,18,37].

There are several limitations of this survey. First, these is potential misclassification of the new and previously treated cases when some cases registered as new but may actually have had TB treatment in the past. Second, the burden of XDR-TB was underestimated because only resistance to ofloxacin and kanamycin not resistance to capreomycin or other aminoglycosides. Third, classification was based on patient history of prior treatment for TB and reviewing of medical records (which were not available for all patients enrolled). Fourth, this survey did not collect information on HIV infection status because patients with tuberculosis in China are not routinely tested for HIV.

## Conclusions

This is the first prevalence survey on resistance to the six major anti-tuberculosis drugs in Lianyungang city. Our study showed that the high prevalence of drug resistance is a major challenge for TB control. Patients with previous treatment history and female gender are risk factors for MDR-TB. Prevention and control of drug-resistant TB should be emphasized by the revised DOTS program through prompt case detection as well as routine and quality-assured DST.

## Competing interests

The authors declare that they have no competing interests.

## Authors’ contributions

QL, LZ, WL conceived the study, analyzed the data and drafted the manuscript; YS, HS, GL, JS participated in the study design, implemented the field investigation and performed DST tests; YZ, CZ and CC participated in the study design and helped draft the manuscript. All authors contributed to the study and have read and approved the final manuscript.

## Pre-publication history

The pre-publication history for this paper can be accessed here:

http://www.biomedcentral.com/1471-2458/13/1171/prepub
